# Aneuploid subtypes of circulating tumor cells and circulating tumor-derived endothelial cells predict the overall survival of advanced lung cancer

**DOI:** 10.3389/fonc.2023.829054

**Published:** 2023-05-05

**Authors:** Jie Zhang, Yang Hong, Liang Wang, Weiheng Hu, Guangming Tian, Di Wu, Yang Wang, Ling Dai, Ziran Zhang, Yue Yang, Jian Fang

**Affiliations:** ^1^ Department of Thoracic Oncology II, Key Laboratory of Carcinogenesis and Translational Research (Ministry of Education), Peking University Cancer Hospital and Institute, Beijing, China; ^2^ Department of Anesthesiology, China-Japan Friendship Hospital, Peking University China-Japan Friendship School of Clinical Medicine, Beijing, China; ^3^ Department of Thoracic Surgery II, Key Laboratory of Carcinogenesis and Translational Research (Ministry of Education), Peking University Cancer Hospital and Institute, Beijing, China

**Keywords:** advanced lung cancer, circulting tumor cells, circulating tumor endothelial cells, prognosis, biomarker

## Abstract

**Objective:**

This study aimed to detect circulating tumor cells (CTCs) and circulating tumor-derived endothelial cells (CTECs) in patients with advanced lung cancer, for describing the distribution characteristics of CTC and CTEC subtypes, exploring the correlation between CTC/CTEC subtypes and novel prognostic biomarkers.

**Methods:**

A total of 52 patients with advanced lung cancer were enrolled in this study. Using the subtraction enrichment-immunofluorescence *in situ* hybridization (SE-iFISH) system, CTCs and CTECs derived from these patients were identified.

**Results:**

Based on cell size, there were 49.3% small and 50.7% large CTCs, and 23.0% small and 77.0% large CTECs. Triploidy, tetraploidy, and multiploidy varied in the small and large CTCs/CTECs. Besides these three aneuploid subtypes, monoploidy was found in the small and large CTECs. Triploid and multiploid small CTCs and tetraploid large CTCs were associated with shorter overall survival (OS) in patients with advanced lung cancer. However, none of the CTECs subtypes showed a significant correlation with patient prognosis. In addition, we found strong positive correlations (P<0.0001) in the four groups including triploid small cell size CTCs and multiploid small cell size CTECs, and multiploid small cell size CTCs and monoploid small cell size CTECs. Furthermore, combined detection of the specific subtypes, including triploid small CTC and monoploid small CTEC, triploid small CTC and triploid small CTEC, and multiploid small CTC and monoploid small CTEC, were associated with poor prognosis in advanced lung cancer.

**Conclusions:**

Aneuploid small CTCs are associated with the outcome of patients with advanced lung cancer. In particular, the combined detection of triploid small CTCs and monoploid small CTECs, triploid small CTCs and triploid small CTECs, and multiploid small CTCs and monoploid small CTECs has clinical significance for predicting prognosis in patients with advanced lung cancer.

## 1 Introduction

The global incidence of lung cancer is increasing, and it has become the leading cause of cancer-related deaths. Approximately 2.09 million new cases and 1.76 million deaths occur from lung cancer each year (1). However, early screening for lung cancer remains challenging, with 57% of lung cancers diagnosed when cancer metastasizes outside the lung (1–3). Despite substantial development in the oncological management of late-stage lung cancer, its prognosis remains poor. The five-year survival at all stages of non-small cell lung cancer (NSCLC) is only 19% (4, 5). The five-year survival rate of patients at different stages varies greatly: 68%-92% for stage I patients, 53%-60% for stage II patients, 13%-36% for stage III patients, and only 0%-10% for stage IV patients (6). Therefore, new strategies for prognosis assessments of advanced NSCLC are urgently needed.

To date, various biomarkers have been found to play important roles in diagnosis, relapse prediction, and drug resistance evaluation in advanced lung cancer, including circulating tumor cells (CTCs), cfDNA, extracellular matrix-associated components, soluble immunological biomarkers, miRNAs, tumor mutation burden, and genetic markers (7–13). CTCs are one of the most prominent biomarkers in the dynamic assessment of cancers and have been used by the FDA in prognostic cancer assessments since 2004. CTCs, which are considered to spread from the tumor into the peripheral blood, leading to metastasis (14), have been studied by researchers worldwide. Characterization of CTCs is considered to be closely related to emerging tumor subclones, influencing treatment response and prognosis. Recently, Kong et al. tested the genomic heterogeneity of CTC in lung and breast cancer and found CTCs genes are more similar to the metastatic tumor compared with the primary tumor (15). Lim et al. found that intratumor heterogeneity of CTCs predicted the risk of recurrence in NSCLC (16). These two studies revealed the significance of CTC heterogeneity in tumor metastasis and prognosis. Furthermore, many researchers have reported the importance of CTCs as biomarkers for prognosis, diagnosis, and drug resistance in cancer (17–22).

With the continuous progress of detection technology, various”cellular circulating tumor markers”, such as circulating tumor endothelial cells (CTECs), have been identified and require further research (17). CTECs are identified as being CD31^+^, where CD31^+^ is widely used to detect endothelial cells, whereas CTCs are identified as being CD31^-^ (23). CTECs decrease in number after operations in esophageal and lung cancers, correlating with reductions in tumor growth (24). In addition, Lei et al. reported that the combined detection of specific CTC and CTEC heteroploid subtypes significantly helped with obtaining higher sensitivity and specificity in identifying malignant nodules in patients with early-stage NSCLC (25). As an important heterogeneity of tumor cells identified by chromosome, aneuploids are considered to play an important role in CTC and CTEC studies (26, 27). Aneuploid quantification of CTCs is a useful tool for tumor progression and metastasis, and the prediction and evaluation of therapeutic efficacy (28). For instance, Ye et al. found that triploid and small CTCs were more aggressive in liver cancer (28); while Li et al. stated that the different ploidies of chromosome 8 were closely related to both sensitivity and resistance to paclitaxel- or cisplatin-based chemotherapy in advanced gastric cancer patients (29). Lin et al. also discussed the significant versatile cellular role of aneuploid CTECs in tumor neovascularization and cancer metastasis (30). Furthermore, several studies have demonstrated that aneuploid CTCs and CTECs may exhibit a functional interplay in tumor angiogenesis, progression, metastasis, and response to therapy (31)—an important novel direction.

In this study, we enrolled patients with late‐stage lung cancer. We aimed to identify CTCs and CTECs in the peripheral blood, conduct subclass analyses of CTCs and CTECs, find a correlation between CTC and CTEC subtypes, and search for prognostic biomarkers.

## 2 Materials and methods

### 2.1 Patient enrollment and specimen collection

A total of 52 patients who were diagnosed with advanced lung cancer between June 2019 and October 2019, and underwent various treatments at Peking University Cancer Hospital were enrolled in this study. Eligibility criteria for patient recruitment included: (1) histological confirmation as lung cancer; (2) being considered as having stage IIIA to IV lung cancer; (3) availability of complete basic information, including age, sex, histology data, TNM stage, and follow-up data; and (4) no diagnosis of any other severe diseases. The exclusion criteria were as follows: (1) having a history of malignancies other than lung cancer within the last five years. Peripheral blood samples (7.5 mL) were collected from the 52 patients before treatment. After the blood samples were collected, they were processed within 24 hours. Each patient provided written informed consent, and this study was approved by the Institutional Ethics Committee of Peking University Cancer Hospital (IRB approval number: 2020KT65). This study was conducted in accordance with the principles of the Declaration of Helsinki.

### 2.2 Subtraction enrichment of CTC and CTEC

To identify CTCs and CTECs, we performed subtraction enrichment and immunostaining fluorescence *in situ* hybridization (SE-iFISH) on the samples. All the experiments were performed in accordance with the manufacturer’s instructions and investigators’ modifications. Cell enrichment was performed using the subtraction enrichment method. A 7.5mL blood sample was centrifuged at 600 × *g* for 5 min, and all the deposited cells were immediately loaded onto 3mL of anon-hematopoietic cell separation matrix (Cytelligen, San Diego, CA, USA). The abovementioned mixture was then centrifuged again at 400 × *g* for 5 min, depleting the red blood cells. Next, the abovementioned supernatants were incubated with anti-leukocyte antibody (CD45) immunomagnetic beads at 25°C for 15 min, and the separation matrix was used again, followed by centrifuging at 400 × *g* for 5 min. Subsequently, the solution was magnetically separated, and the magnetic beads were then removed from the supernatant. The bead-free solution was centrifuged at 500 × *g* for 2 min, and the cells were mixed thoroughly with 100μL of cell fixative. Finally, the cell mixture was smeared on coated CTC slides and dried overnight for subsequent iFISH processing.

### 2.3 iFISH

The samples thus obtained were then processed according to the manufacturer’s instructions and investigators’ modifications (Cytelligen, San Diego, CA, USA). The samples were subjected to the Vysis Centromere Probe (CEP8) Spectrum Orange (Abbott molecular, Abbott Park, Illinois,USA) for 4 hours, followed by incubation with Alexa Fluor 594-conjugated monoclonal anti-CD45 antibodies (Cytelligen, San Diego, CA, USA) and Cy5-conjugated monoclonal anti-CD31 antibodies (Cytelligen, San Diego, CA, USA) at 1: 200 dilution for 30 minutes at room temperature. Finally, 4-6-diamidino-2-phenylindole (DAPI; Life Technologies, Carlsbad,California, USA) was used to stain the nuclei. Stained cells were observed and counted under a fluorescence microscope. At least two pathologists performed CTC and CTEC counting for DAPI^+^, CD45^-^, and CD31^-/+^ cells, identified chromosome 8 aneuploidy under fluorescence, and conducted subclasses according to cell size and ploidy. Based on our previous study (32), CTCs or CTECs ≤5 µm in size (approximately the size of a white blood cell [WBC] or less) were considered small cell size CTCs or CTECs, whereas those>5 µm in size were considered large cell size CTCs or CTECs.

### 2.4 Isolation and identification of aneuploid CTCs and CTECs

**Figure 1:**
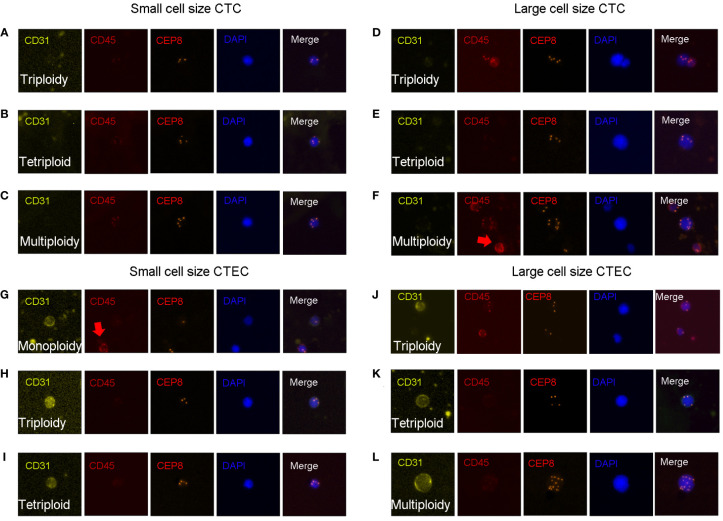
CTC and CTEC detection. Image representations of different sizes and ploidies of CTCs/CTECs from patients with advanced lung cancer. **(A–F)** CTCs are DAPI+/CD45-/CD31-/CEP8+. **(G–L)** CTECs are DAPI+/CD45-/CD31+/CEP8+. **(A–C)** Small CTCs including triploid small CTC **(A)**, tetraploid small CTC **(B)**, and multiploid small CTC **(C)**. **(D–F)** Large CTCs including triploid large CTCs **(D)**, tetraploid large CTCs **(E)**, and multiploid large CTCs **(F)**. **(G–I)** Small CTECs including monoploid small CTECs **(G)**, triploid small CTECs **(H)**, tetraploid small CTECs **(I)**. **(J–L)** Large CTECs including triploid large CTECs **(J)**, tetraploid large CTECs **(K)**, and multiploid large CTECs **(L)**.

CTC identification criteria were as follows (**Figures 1A–F**): DAPI^+^, CD45^-^, CD31^-^, and the identification of chromosome 8 aneuploidy. CTEC identification criteria were as follows (**Figures 1G–L**): DAPI^+^, CD45^-^, CD31^+^, and the identification of chromosome 8 aneuploidy.

With the detection of chromosomal 8 aneuploidy and cell size, we could achieve further subclassification of CTCs and CTECs. Using the general size of WBCs as the threshold, CTCs/CTECs were identified as either small (≤ 5 µm; **Figures 1A–C, G-I**) or large (>5 µm; **Figures 1D–F, J–L**). On detection of chromosomal 8 aneuploidy, CTCs were further divided into triploid (Figures A, D), tetraploid(Figures B, E), and multiploid (Figures C, F) subtypes, and CTECs were further divided into monoploid (Figure G), triploid (Figures H, J), tetraploid (Figures I, K), and multiploid (Figure L) subtypes.

### 2.5 Statistical analyses

All statistical analyses were performed using GraphPad Prism 7.0 and IBM SPSS Statistics software version 23.0. Correlations between CTCs and CTECs were calculated and analyzed using chi-square tests. Overall survival (OS) was defined as the duration from the treatment initiation till death. Kaplan-Meier survival plots for OS were generated based on whether CTC/CTEC numbers were more or less than the median of CTC/CTEC numbers. Log-rank tests were used to compare survival curves, and hazard ratio (HR) values were also shown at the same time. The possible significant predictors of OS were then enrolled into a multivariable Cox regression Model, identifying independent significant predictors of OS. All P values were two-sided, and P < 0.05 was defined as statistically significant.

## 3 Results

### 3.1 Distribution of CTC and CTEC subtypes in the advanced lung cancer patients

The study included 52 patients with advanced lung cancer. The characteristics of the patients are presented in **Table 1**. In total, 37 (71%) male and 15 (29%) female patients were included in this study, with a median age of 63 years, and an average age of 63 years (ranging from 36–78 years). For the pretreatment clinical stage, nine (17.3%) and 43 (82.7%) patients were stage III and IV, respectively.

**Table 1:** Characteristics of patients (n=52).

Variations	Variable	No. of cases	Percentage (%)
Age	≤60	19	36.5
	>60	33	63.5
Sex	Male	37	71.2
	Female	15	28.8
Smoking	No	19	36.5
	Yes	33	63.5
Histology	LUSC	31	59.6
	LUAD	16	30.8
	Other types	5	9.6
Pre-treatment clinical TNM stage	III	9	17.3
	IV	43	82.7

LUSC, lung squamous cell carcinoma; LUAD, lung adenocarcinoma.

**Figure 2:**
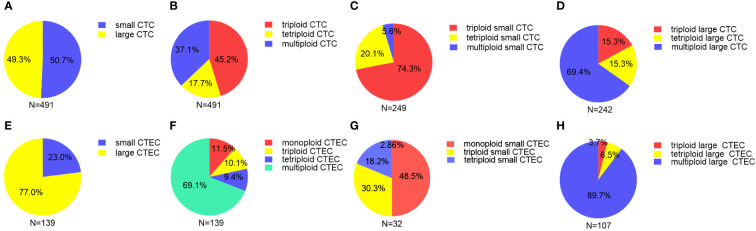
Proportion of different sizes and aneuploid CTCs/CTECs **(A)** Proportion of small and large CTCs. **(B)** Proportion of heteroploid subtypes of total CTCs. **(C)** Proportion of heteroploid subtypes of small CTCs. **(D)** Proportion of heteroploid subtypes of large CTCs. **(E)** Proportion of small and large CTECs. **(F)** Proportion of heteroploid subtypes of total CTECs. **(G)** Proportion of heteroploid subtypes of small CTECs. **(H)** Proportion of heteroploid subtypes of large CTECs.

In our study of 52 patients, we found 491 CTCs in our patient cohort, including 249 (50.7%) small CTCs and 242(49.3%) large CTCs (**Figure 2A**). In addition, total, small, and large CTCs were detected in 90.4% (47/52), 84.6% (44/52), and 75% (39/52) of the patients, respectively (**Table 2**). The heteroploid features of CTCs are shown in **Figures 2B–D**. Triploidy accounted for the largest proportion of small CTCs (74.3%), followed by tetraploidy (20.1%) and multiploidy (5.6%); however, multiploidy accounted for the largest proportion of large CTCs (69.4%), followed by tetraploidy (15.3%) and triploidy (15.3%).

**Table 2:** CTC and CTEC detection (n=52).

Variation	Number of CTCs (%)			Number of CTECs (%)		
	Total	small cell size CTC	large cell size CTC cases	Total	small cell size CTEC	large cell size CTEC cases
Monoploidy	0 (0%)	0 (0%)	0 (0%)	5 (9.6%)	5 (9.6%)	0 (0%)
Triploidy	40 (76.9%)	39 (75%)	15 (28.8%)	10 (19.2%)	6 (11.5%)	4 (%)
Tetraploidy	33 (63.5%)	22 (42.3%)	23 (44.2%)	12 (23.1%)	5 (9.6%)	7 (13.5%)
Multiploidy	37 (71.2%)	8 (15.4%)	36 (69.2%)	29 (55.8%)	0 (0%)	29 (55.8%)
Total	47 (90.4%)	44 (84.6%)	39 (75%)	35 (67.3%)	12 (23.1%)	30 (57.7%)

As for CTECs, we found 139 CTECs in our patient cohort, including 32 (23.0%) small CTECs and 107 (77%) large CTECs (**Figure 2E**). Total, small, and large CTECs were detected in 67.3% (35/52), 23.1% (12/52), and 57.7% (30/52) of patients, respectively (**Table 2**). Monoploidy accounted for the highest proportion of small CTECs (48.5%); however, multiploidy accounted for the highest proportion of large CTECs (89.7%) (**Figures 2F–H**).

### 3.2 CTC/CTEC subtypes and OS

**Figure 3:**
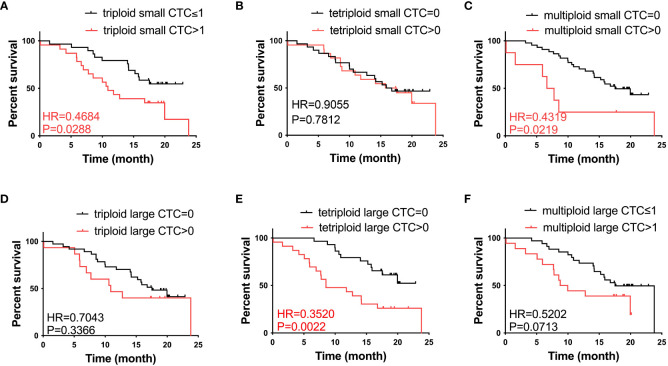
Survival analysis. **(A)** Patients with triploid small CTCs>1 had shorter OS compared to those with triploid CTCs ≤ 1 (P < 0.05). **(B)** Tetraploid small CTC subtypes were not correlated with OS (P > 0.05). **(C)** Patients with multiploid small CTCs>0 had shorter OS compared to those with multiploid CTCs=0 (P < 0.05). **(D)** Triploid large CTC subtype was not correlated to OS (P > 0.05). **(E)** Patients with tetraploid large CTCs>0 had shorter OS compared to those with tetraploid CTCs=0 (P < 0.05). **(F)** Multiploid small CTC subtype was not correlated to OS (P > 0.05).

To further investigate the role of CTCs in prognosis, we analyzed the correlation between the heteroploid subtypes of small/large cell size CTCs and OS in patients with advanced lung cancer. Based on the median value of CTCs, we found that patients with triploid small CTCs>1, multiploid small CTCs>0, and tetraploid large CTCs>0 had shorter OS than patients with triploid small CTCs ≤ 1, multiploid small CTCs=0 and tetraploid large CTCs=0 (**Figures 3A, C, E**); However, patients with tetraploid small CTC>0, triploid large CTC>0, and multiploid large CTC>1 had no differences with tetraploid small CTCs=0, triploid large CTCs=0, and multiploid large CTCs ≤ 1 (**Figures 3B, D, F**). In summary, CTC heteroploidy subtypes that were detected using the SE-iFISH system, including triploid and multiploid small CTCs, together with tetraploid large CTCs, correlated with the prognosis of advanced lung cancer.

**Figure 4:**
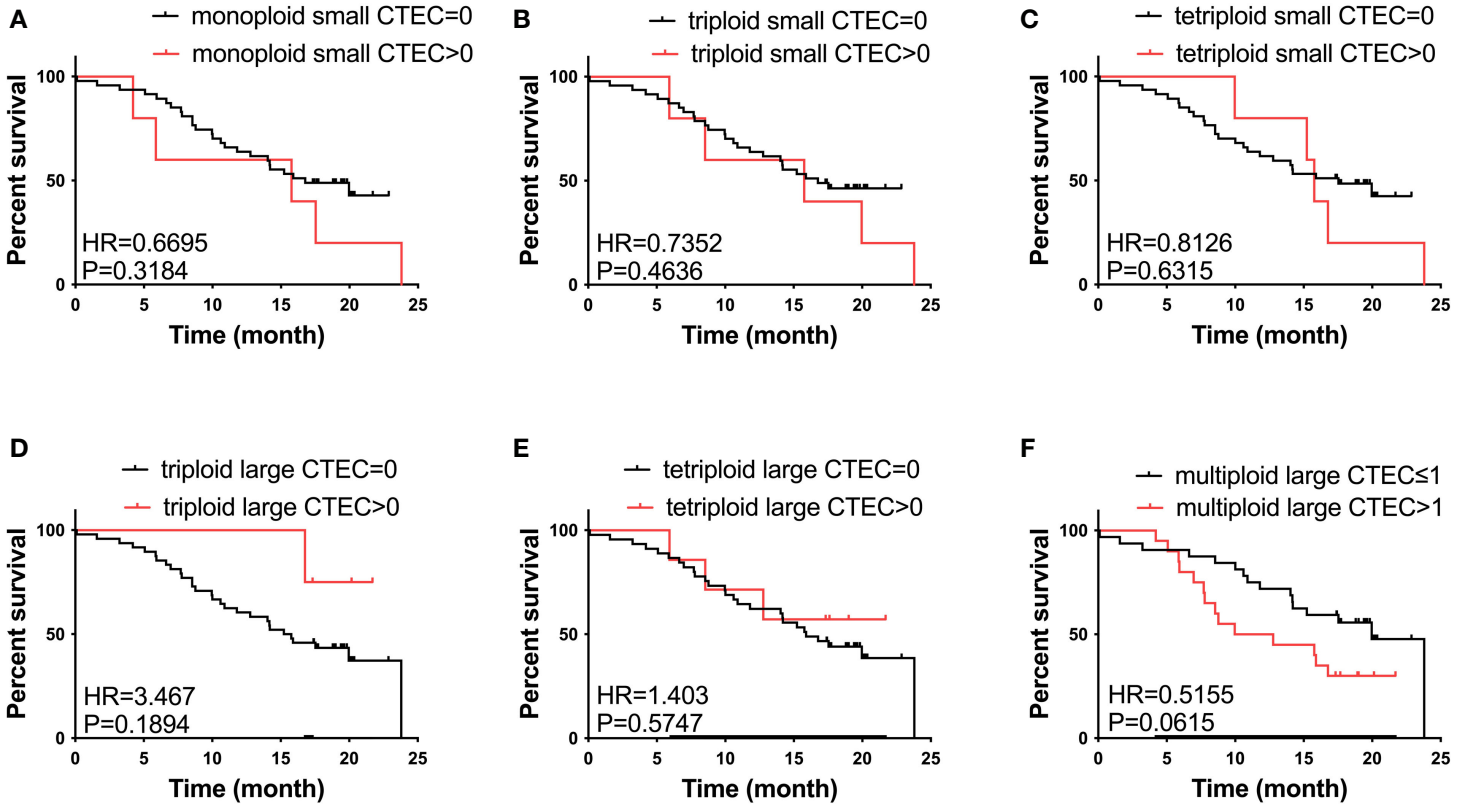
Survival analysis. **(A–F)** No CTEC subtypes were correlated to the OS of patients with advanced NSCLC (P > 0.05).

We also studied the relationship between CTEC subtypes and OS in patients with advanced lung cancer. However, none of the aneuploid CTEC subtypes were significantly related to OS (**Figures 4A–F**).

### 3.3 Relationships between numbers of CTC and CTEC subtypes

**Figure 5:**
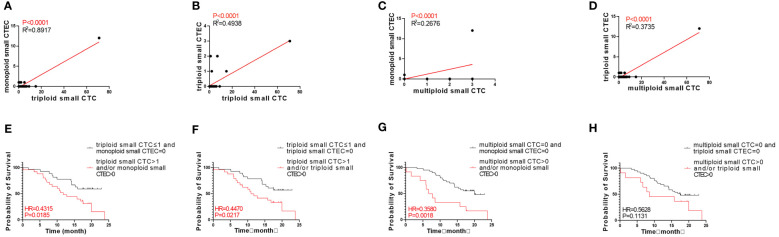
Correlation between CTC and CTEC subtypes, and survival of combined detection of CTC and CTEC subtypes with univariable analyses. **(A–D)** Correlations in four groups including triploid small CTC and monoploid small CTEC **(A)**, triploid small CTC and triploid small CTEC **(B)**, multiploid small CTC and monoploid small CTEC **(C)**, and multiploid small CTC and triploid small CTEC **(D)**. **(E–H)** Survival analyses in four groups including triploid small CTC≤1 and monoploid small CTEC=0 vs. triploid small CTC> 1 and/or monoploid small CTEC>0 **(E)**, triploid small CTC≤1 and triploid small CTEC=0 vs. triploid small CTC> 1 and/or triploid small CTEC>0 **(F)**, multiploid small CTC=0 and monoploid small CTEC=0 vs. multiploid small CTC>0 and/or monoploid small CTEC>0 **(G)**, multiploid small CTC=0 and triploid small CTEC= 0 v s . multiploid small CTC>0 and/or triploid small CTEC> 0 **(H)**.

Next, we analyzed whether the CTC subtypes associated with OS (**Figures 3A, C, E**) were correlated with the CTEC subtypes in advanced lung cancer. As shown in **Table 3**, four groups, including triploid small cell size CTCs and monoploid/triploid small cell size CTECs, and multiploid small cell size CTCs and monoploid/triploid small cell size CTECs, had extremely strong positive correlations (P<0.0001). The relationships between these CTC and CTEC subtypes (P<0.0001) are described in **Figure 5A–D**.

**Table 3:** Correlations between CTC subtypes and CTEC subtypes in advanced lung cancer.

		Triploid small cell size CTC	Multiploid small cell size CTC	Tetraploid large CTC
Small cell size CTEC	Monoploid	****	****	NS
	Triploid	****	****	NS
	Tetraploid	*	NS	NS
	Multiploid	NS	NS	NS
Large cell size CTEC	Monoploid	NS	NS	NS
	Triploid	NS	NS	NS
	Tetraploid	NS	NS	*
	Multiploid	NS	NS	NS

****P<0.0001, ***P<0.001, **P<0.01, *P<0.05, NS, P>0.05.

Based on the abovementioned results, we examined whether the combined detection of CTC and CTEC subtypes is important in determining the prognosis associated with advanced lung cancer. The combined detection of triploid small CTCs and monoploid small CTECs, triploid small CTCs and triploid small CTECs, and multiploid small CTCs and monoploid small CTECs (P<0.05) could also predict prognosis in advanced lung cancer (**Figures 5E–H**). Patients with triploid small CTCs>1 and/or monoploid small CTECs>0 had shorter OS than did patients with triploid small CTCs ≤ 1 and monoploid small CTECs=0 (P=0.0185, **Figure 5E**). Patients with triploid small CTCs>1 and/or triploid small CTECs>0 had shorter OS than did patients with triploid small CTCs ≤ 1 and triploid small CTECs=0 (P=0.0217, **Figure 5F**). Patients with multiploid small CTCs>0 and/or monoploid small CTECs>0 had shorter overall survival than patients with multiploid small CTCs=0 and monoploid small CTECs=0 (P=0.0018, **Figure 5G**). However, OS of patients with multiploid small CTCs>0 and/or triploid CTECs>0 was not different from that of patients with multiploid small CTCs=0 and triploid small CTEC s=0 (P=0.1131, **Figure 5H**). Furthermore, we noticed that these three combined detections had a better effect in predicting prognosis, which had smaller p-values than did detecting small CTC subtypes independently (**Table 4**). Taking the combined detection of triploid small CTCs and monoploid small CTECs for instance, the p-value for detecting triploid small CTCs independently was 0.0288; however, the p-value for the combined detection of triploid small CTCs and monoploid small CTECs was only 0.0185, indicating better detection efficiency of the combined detection.

**Table 4:** Survival analyses of CTC subtypes, CTEC subtypes, and combinations of CTC and CTEC subtypes.

Triploid small CTC			Monoploid small CTEC			Combined detection		
P values	Mean Survival Time(m)		P values	Mean Survival Time(m)		P values	Mean Survival Time(m)	
	Triploid small CTC ≤1	Triploid small CTC >1		Monoploid small CTEC=0	Monoploid small CTEC >0		Triploid small CTC≤1 and monoploid small CTEC=0	Triploid small CTC>1 and/or monoploid small CTEC>0
**0.0288**	17.712	12.920	**0.3184**	15.847	13.434	**0.0185**	17.817	13.168
Triploid small CTC			Triploid small CTEC			Combined detection		
P values	Mean Survival Time(m)		P values	Mean Survival Time(m)		P values	Mean Survival Time(m)	
	Triploid small CTC≤1	Triploid small CTC >1	Triploid small CTEC=0	Triploid small CTEC >0	Triploid small CTC≤1 and triploid small CTEC=0	Triploid small CTC>1 and/or triploid small CTEC>0		
**0.0288**	17.712	12.920	**0.8224**	15.638	15.981	**0.0217**	17.781	13.038
Multiploid small CTC			Monoploid small CTEC			Combined detection		
P values	Mean Survival Time(m)		P values	Mean Survival Time(m)		P values	Mean Survival Time(m)	
	Multiploid small CTC=0	Multiploid small CTC >0	Monoploid small CTEC=0	Monoploid small CTEC >0	Multiploid small CTC=0 and monoploid small CTEC=0	Multiploid small CTC>0 and/or monoploid small CTEC>0		
**0.0219**	16.684	9.766	**0.3184**	15.847	13.434	**0.0018**	17.294	10.125
Multiploid small CTC			Triploid small CTEC			Combined detection		
P values	Mean Survival Time(m)		P values	Mean Survival Time(m)		P values	Mean Survival Time(m)	
	Multiploid small CTC=0	Multiploid small CTC >0	Triploid small CTEC=0	Triploid small CTEC>0	Multiploid small CTC=0 and triploid small CTEC=0	Multiploid small CTC>0 and/or triploid small CTEC>0		
**0.0219**	16.684	9.766	**0.8224**	15.638	15.981	**0.1131**	16.594	12.167

### 3.4 Combined detection of CTCs and CTECs by multivariable Cox regression analyses for OS

**Figure 6:**
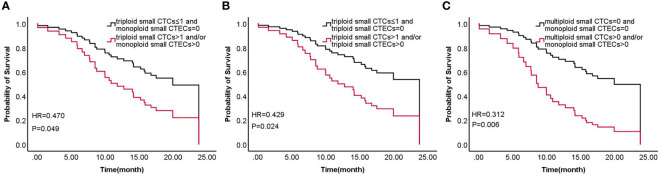
Survival of combined detection of CTC and CTEC subtypes multivariable cox regression analyses in three groups **(A–C)** including triploid small CTCs≤1 and monoploid small CTECs=0 vs. triploid small CTCs>1 and/or monoploid small CTECs>0 **(A)**, triploid small CTCs≤ 1 and triploid small CTECs=0 vs. triploid small CTCs>1 and/or triploid small CTECs>0 **(B)**, and multiploid small CTCs=0 and monoploid small CTECs=0 vs. multiploid small CTCs>0 and/or monoploid small CTECs>0 **(C)**.

According to the results, the combined detections of three groups (Group 1: triploid small CTCs ≤ 1 and monoploid small CTECs=0 vs. triploid small CTCs>1 and/or monoploid small CTECs>0; Group 2: triploid small CTCs ≤ 1 and triploid small CTECs=0 vs. triploid small CTCs>1 and/or triploid small CTECs>0; and Group 3: multiploid small CTCs=0 and monoploid small CTECs=0 vs. multiploid small CTCs>0 and/or monoploid small CTECs>0) showed a significant difference in OS by univariable analysis. Furthermore, we analyzed these groups in OS by the multivariable Cox regression analyses, revealing that combined detection of group 1 (hazard ratio: 0.47, 95% CI: 0.225-0.981; P<0.05), group 2 (hazard ratio: 0.429, 95% CI 0.204-0.903, P<0.05), and group 3 (hazard ratio: 0.312, 95% CI: 0.144-0.676, P<0.05) were significant independent predictors for longer OS, respectively (**Table 5**; **Figure 6**).

**Table 5:** Multivariable cox regression analyses for OS in the groups of combinational CTC and CTEC subtypes.

Patient Variable		Multivariable model	
		HR (95% CI)	P value
Sex	Female		
	Male		
Age	≤60		
	>60		
Smoking	No		
	Yes		
TNM Stage	III		
	IV		
Group 1		0.470(0.225-0.981)	0.049
Group 2		0.429(0.204-0.903)	0.024
Group 3		0.312(0.144-0.676)	0.006

Group 1 triploid small CTCs≤1 and monoploid small CTECs=0 vs. with triploid small CTCs>1 and/or monoploid small CTECs>0; Group 2: triploid small CTCs≤1 and triploid small CTECs=0 vs. triploid small CTCs>1 and/or triploid small CTECs>0; Group 3: multiploid small CTCs=0 and monoploid small CTECs=0 vs. multiploid small CTCs>0 and/or monoploid small CTECs>0.

**Figure 7:**
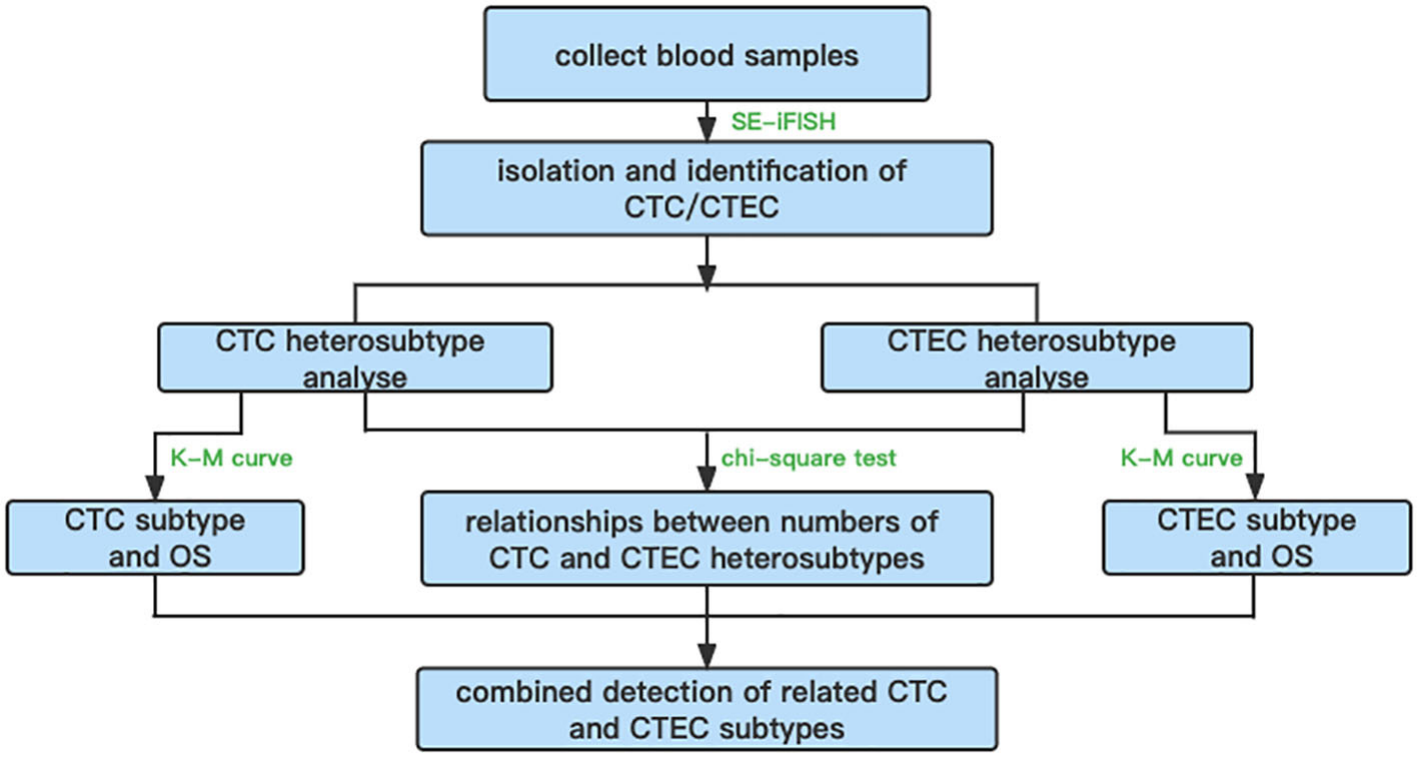
The overall flowchart.

In summary, an overall flowchart of this study is shown in **Figure 7**.

## 4 Discussion

In this study, we explored the relationship between CTC and CTEC subtypes in patients with advanced lung cancer and attempted to identify prognostic biomarkers of this disease. Using the SE-iFISH method, we found that the detection rates of CTCs and CTECs were as high as 90% and 67%, respectively. Regarding the distribution of CTC and CTEC subtypes, we distinguished CTCs and CTECs in two dimensions, namely, heteroploid characteristics of chromosome 8 and cell size. Then, we further studied these CTC/CTEC subtypes and the prognosis of advanced lung cancer patients.

Hiroaki et al. showed that tumor cells undergoing epithelial-to-mesenchymal transition (EMT) were smaller in size than those without EMT characteristics (33). Wang et al. found that the majority of CTCs that were Vimentin^+^ (a marker for mesenchymal cells) were small in size and demonstrated different features from CTCs that were Vimentin^-^ (34). Chen et al. found that the sizes of circulating tumor cell clusters can be used to track therapeutic resistance and prognosis in advanced gastric cancer (35). PD-L1^+^ aneuploid CTECs could exhibit resistance to immunotherapy in advanced NSCLC (31). In addition, the size of CTCs and clinical characteristics, such as cancer metastasis and progression (36), and post-surgical recurrence (37) were found to be relevant. These studies revealed that small cell size CTCs with more malignant behavior are associated with tumor progression and poor prognosis. Therefore, in this study, we did a subclassification of CTCs/CTECs into small cell size (≤white blood cell (WBC)) and large cell size (>WBC). We found that small CTCs accounted for 50.7% of the total CTCs, while large CTCs accounted for 49.3%; however, small CTECs accounted for only 23% of the total CTECs, and large CTECs accounted for 77% of the total CTECs.

Through the CellSearch system, Krebs et al. found 21% of positive CTCs (at baseline of > 2 CTCs) in the total cases of lung cancer patients (n=101) with IIIa to IV stages lung cancer and 32% of positive CTCs in NSCLC cases with stage IV lung cancer (n=60) (38). With the CellSearch system, Juan et al. also used two CTCs as a baseline and found that 24% of patients (n=37) had positive CTCs in the NSCLC patients who received chemotherapy (39). Using the SE-iFISH method, Ge et al. found that 92% of lung cancer patients (24/26) showed positive CTCs at a baseline of one CTC (40); Ye et al. reported that 92.9% (79/85) of lung cancer patients had positive CTCs (28). In our previous study, 88% and 94% of patients with resectable NSCLC were positive for total circulating aneuploid cells (CACs) in pre- and post-surgery, respectively (32). In the present study, we found that CTCs were identified in 90.4% (47/52) of lung cancer patients with advanced stage. Since the heterogenicity and high frequency of CTCs in these lung cancers, we analyzed the role of the size and aneuploid subtypes of CTCs/CTECs in the prognosis of these patients. In our previous study on early-stage lung cancer, small CACs accounted for only 18% of the total CACs (32). Furthermore, in our study on advanced lung cancer, small CTCs accounted for 50.7% of all CTCs. In addition, we found that small CTECs only accounted for 23% of all CTECs, and mainly comprised monoploid (16/32) and triploid (10/32) CTECs.

Chromosomal instability mainly leads to chromosome aneuploidy, which is a common feature in solid tumors and the causes of aneuploidy contain kinetochore–microtubule attachments and dynamics, centrosome number, spindle assembly checkpoint, and chromosome cohesion (26, 27). Several studies clarified the relationship between subtypes of aneuploidy CTCs and clinical characteristics, diagnosis, prognosis, and drug resistance (29, 31, 32, 37, 41, 42). Herein, aneuploidy CTCs/CTECs including monoploidy, triploidy, tetraploidy, and multiploidy subtypes were also identified. Triploid and multiploid small CTCs, together with tetraploid large CTCs were found to associate with the prognosis of advanced lung cancer.

CTCs are used in the diagnosis, prognosis evaluation, efficacy evaluation, drug resistance and recurrence monitoring, and precision treatment target screening of various tumors. CTCs have shown important clinical application value and have become a hot topic in tumor research (43–45). In addition, with the continuous development of CTC detection technology, another “cellular circulating tumor marker” (that is, CTECs) has been preliminary studied (30, 31, 42). Among the endothelial cells that constitute the tumor vascular system, most are tumor-derived endothelial cells that express a high level of CD31. These endothelial cells also exhibit cytogenetic abnormalities in aneuploid chromosomes. A previous study on the CTEC karyotype illustrated that normal endothelial cells are strictly diploid, whereas tumor endothelial cells (TECs) contain multiple chromosomal aneuploidies. Tumor-derived endothelial cells, which have the dual characteristics of malignant tumors and an endothelial vascularization ability, are endothelialized cancer cells. These cells enter the circulation from blood vessels to become CTECs, and this migration may play an important role in the formation of new blood vessels in metastatic tumors, thus having important clinical significance (30, 46, 47).

SE-iFISH is a detection technology that can identify target cells from peripheral blood cells by combining the detection of tumor marker expression on the surface of tumor cells and chromosomal aneuploidy. Therefore, comprehensive co-detection of aneuploid CTCs (CD31^-^) and CTECs (CD31^+^) using SE-iFISH was performed simultaneously in our study (25). In this way, high specificity (with respect to detecting CTCs/CTECs by iFISH) was ensured by distinguishing between aneuploidy and specific markers in the target cells. Based on this SE-iFISH system, we found that some CTC subtypes (triploid and multiploid small CTCs and tetraploid large CTCs) could be biomarkers for shorter OS; however, CTEC subtypes did not exhibit this characteristic.

Lin et al. hypothesized that aneuploid CTECs and CTCs cross-talk and influence each other in the circulation, leading to cancer metastasis and progression in some way (30). Lei et al. found that the combined detection of aneuploid CTECs and CTCs could be a good biomarker for the diagnosis of early-stage lung cancer (25). Therefore, we further investigated whether CTC and CTEC subtypes were related in patients with advanced lung cancer. According to our results, Pearson correlation tests confirmed significantly positive correlations in four groups, including triploid small cell size CTCs and monoploid/triploid small cell size CTECs, and multiploid small cell size CTCs and monoploid/triploid small cell size CTECs (P<0.0001). In addition, we found that the combined detection of three pairs of related CTC and CTEC subtypes (including triploid small CTC and monoploid small CTEC, triploid small CTC and triploid small CTEC, and multiploid small CTC and monoploid small CTEC) helped predict poor OS, even better than small CTC detection alone.

Recently, Lin reported that aneuploid TECs are generated from the “cancerization of stromal endothelial cells” and “endothelialization of carcinoma cells” in the hypoxic tumor microenvironment. Both of these processes are deeply involved in hypoxia-triggered epithelial-to-mesenchymal transition (EMT) and endothelial-to-mesenchymal transition (EndoMT) (30), which might be the reason why CTECs play important roles in predicting prognosis. Furthermore, CTECs are TECs that flow into the peripheral circulation, and CTCs have been widely reported to be associated with tumor EMT, which may explain why the numbers of CTCs and CTECs were closely correlated in our study (33, 34).

Unlike single detection of CTCs, synchronous and combined detection of CTCs and CTECs plays an important role in the evaluation of tumor prognosis. In addition, the combined detection of CTCs and CTECs also has research potential in clinical applications, such as tumor diagnosis and real-time monitoring of curative effect prediction and recurrence, which may provide effective technical support for individualized, accurate diagnosis and treatment of patients. However, the role of these aneuploid malignant cells in tumor formation and metastasis requires further study.

## 5 Conclusion

Triploid and multiploid small CTCs are good prognostic biomarkers for advanced lung cancer. Moreover, combined detection of small CTC and small CTEC heteroploid subtypes can predict OS in advanced lung cancer, and it showed better detection efficiency than that of individualized detection alone.

## Data availability statement

The original contributions presented in the study are included in the article/supplementary material. Further inquiries can be directed to the corresponding authors.

## Ethics statement

The studies involving human participants were reviewed and approved by the medical ethics committee of Beijing Cancer Hospital. The patients/participants provided their written informed consent to participate in this study.

## Author contributions

JZ, YH, LW, YY, and JF contributed to conception and design of the study. Medical practitioners, WH, GT, DW, YW, LD, ZZ, YY provided patients and patient data for the study. JZ, YH, LW, and JF organized the database, and JZ, YH, LW, and JF performed the statistical analysis. JZ, YH, LW, and JF supported the literature, statistical methods and the detection method. JZ, YH, LW, and JF wrote the manuscript. JZ, YH, and LW provided correlated images. All authors contributed to the article and approved the submitted version.
